# Supplementary material for: P2X7 receptor inhibition ameliorates dendritic spine pathology and social behavioral deficits in Rett syndrome mice
Source: Nat Commun. 2020 Apr 14;11:1784. doi: 10.1038/s41467-020-15590-5 (PMC7156443; doi:10.1038/s41467-020-15590-5)
Supplement: Supplementary file 1 — Supplementary Information [file 41467_2020_15590_MOESM1_ESM.pdf]

Supplementary Information

P2X7 receptor inhibition ameliorates dendritic spine pathology and social behavioral deficits in Rett syndrome mice

Garré et al.

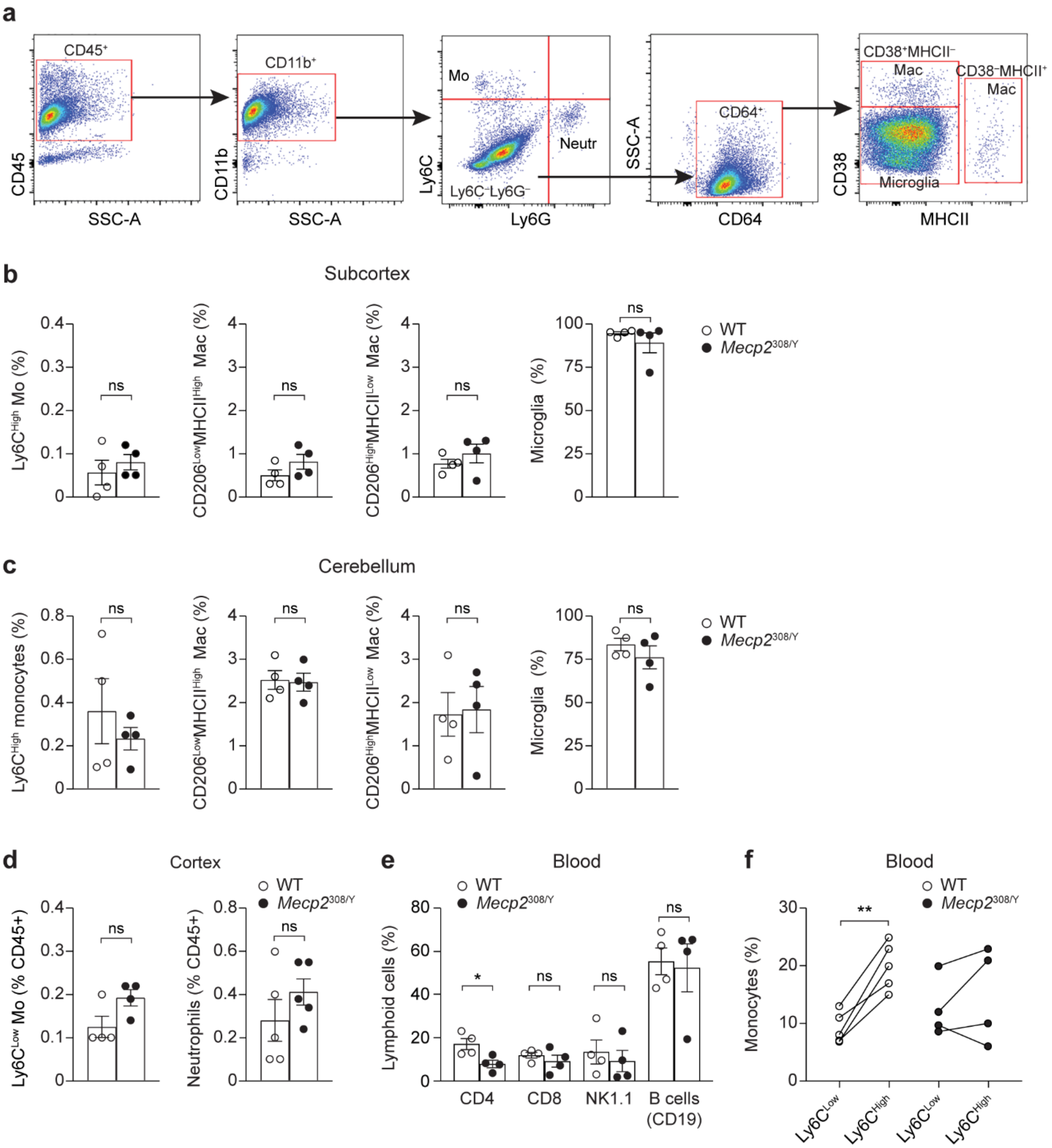

Supplementary Figure 1. Characterization of immune cell populations in cortex, subcortex, cerebellum and blood of *Mecp2*$^{308/Y}$ mice.

a, Representative flow cytometry analysis showing the gating strategy for the analysis of Ly6C^{High} monocytes (Mo), neutrophils (Neutr), microglia and macrophage (Mac) populations. Microglia are CD38$^{–}$MHCII$^{–}$ cells, macrophage subsets include CD38^{+}MHCII$^{–}$ and CD38$^{–}$MCHII^{+} cells. **b** and **c**,

Percentages of Ly6C^{High} monocytes, CD206^{Low}MHCII^{High} and CD206^{High}MHCII^{Low} macrophages and microglia in the subcortex (**b**) and cerebellum (**c**) of WT and *Mecp2*$^{308/Y}$ mice (*n* = 4 mice per group). Data are expressed as percentages of total CD45^{+} cells in each region. **d**, Percentages of Ly6C^{Low} monocytes and neutrophils in the cortex of WT and *Mecp2*$^{308/Y}$ mice (*n* = 4 mice per group). Ly6C^{Low} monocytes were gated on CD11b^{+}CD45^{High}MHCII^{Low}Ly6C^{Low}Ly6G$^{–}$ (P = 0.0746), neutrophils were gated on Ly6C^{Low}Ly6G^{+} (P = 0.2820). Data are expressed as percentages of total CD45^{+} cells in the cortex. **e**, Flow cytometry analysis of lymphoid cells in the blood of WT and *Mecp2*$^{308/Y}$ mice (*n* = 4 mice per group; CD4, P = 0.0251; CD8, P = 0.4048; NK1.1, P = 0.5918; CD19, P = 0.8215). **f**, Flow cytometry analysis of blood monocytes in WT and *Mecp2*$^{308/Y}$ mice (WT, *n* = 5 mice; P = 0.0029; *Mecp2*$^{308/Y}$, *n* = 4 mice, P = 0.4223). Data are presented as mean ± SEM. *P < 0.05, **P < 0.01, unpaired two-tailed *t* test in **b**-**e**, paired two-tailed *t* test in **f**. Source data are provided as a Source Data file.

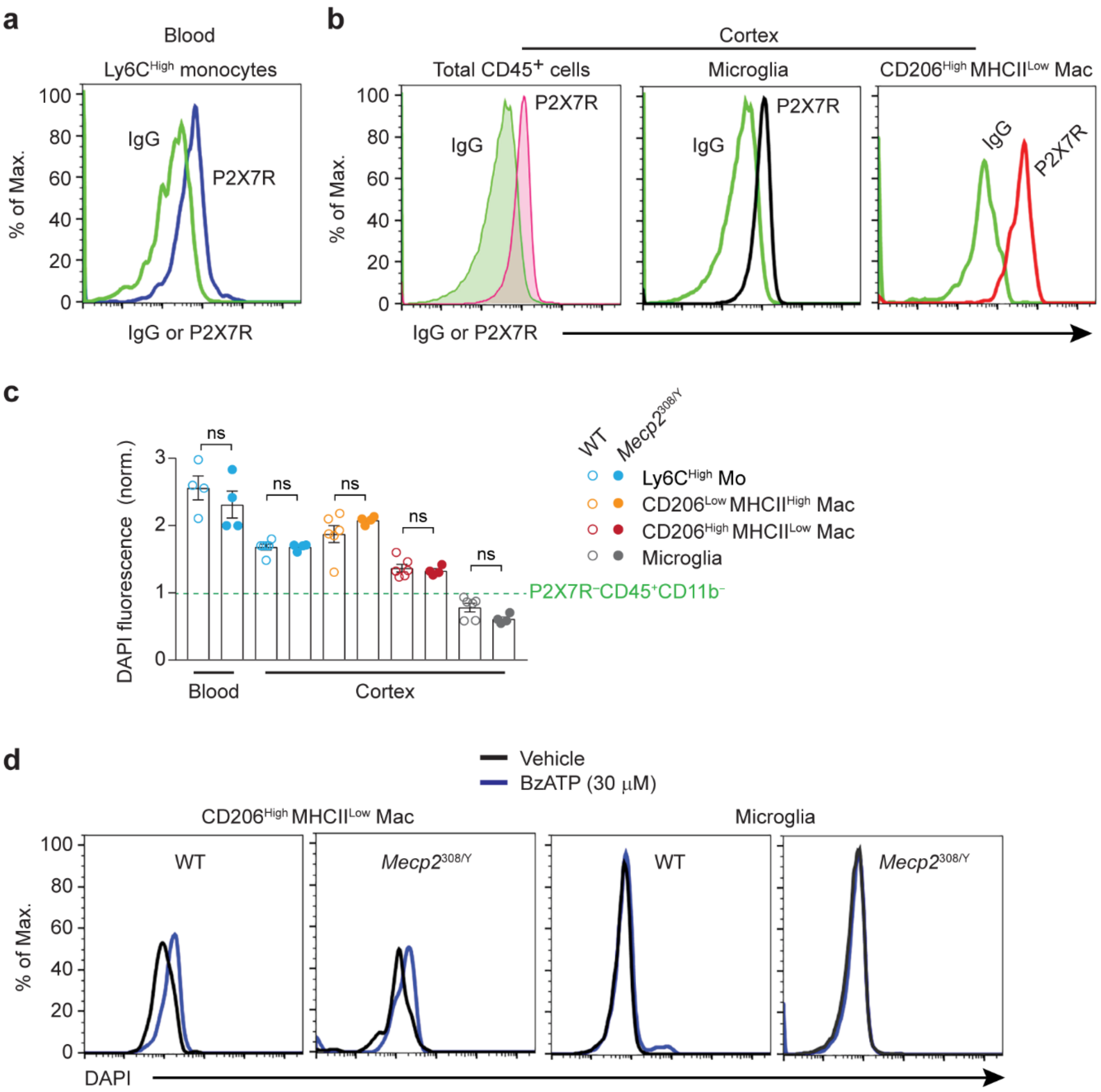

Supplementary Figure 2. P2X7Rs are abundantly expressed in monocytes and macrophages.

a, Histogram showing distribution of surface P2X7R expression in circulating Ly6C^{High} monocytes. Fluorescence intensity of cells incubated with an anti-P2X7R antibody are compared to cells incubated with an IgG isotype, as a negative control. **b**, Same strategy as in **a** but showing distribution of surface P2X7Rs in total CD45^{+} cells, microglia and CD206^{High}MHCII^{Low} macrophages (Mac) in the cortex. **c**, Baseline DAPI labeling in P2X7R^{+} blood and cortical leukocytes is not different between WT and *Mecp2*$^{308/Y}$ mice. DAPI fluorescence in P2X7R^{+} leukocytes is normalized to that in P2X7R^{-} cells. DAPI fluorescence in monocytes (Mo), macrophages and microglia overlaps with the background fluorescence (unstained control)

measured in each cell type, indicating negligible baseline dye uptake in both WT and *Mecp2*$^{308/Y}$ (n = 4 mice per group). **d**, CD206^{High}MHCII^{Low} macrophages (P2X7R^{High}) increase DAPI uptake in response to ATP. Histograms showing distribution of DAPI fluorescence intensity in WT and *Mecp2*$^{308/Y}$ microglia and CD206^{High}MHCII^{Low} macrophages after 15 min of vehicle or Benzoyl ATP (BzATP; 30 μM) treatment. BzATP is a P2X7R agonist. Data are representative of 2 independent experiments (n = 3). Data are presented as mean ± SEM. ns, not significant, unpaired two-tailed t test was used in **c**. Source data are provided as a Source Data file.

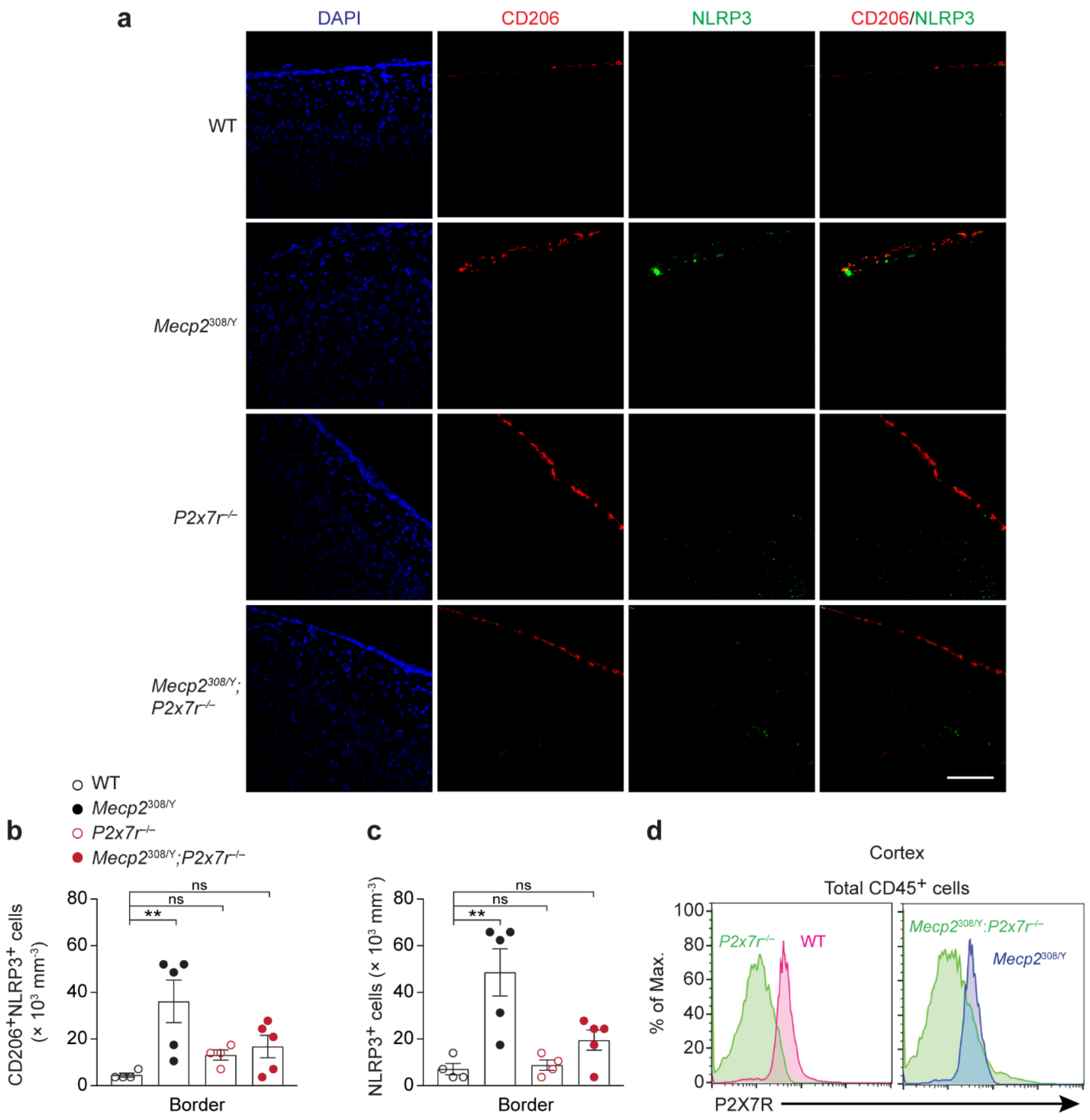

Supplementary Figure 3. P2X7 receptors mediate inflammasome activation in CD206⁺ macrophages in *Mecp2*$^{308/Y}$ mice.

a, Representative coronal sections of the mouse cortex stained for NLRP3 (green) and CD206 (red). Cell nuclei were colored with DAPI (blue). NLRP3 was primarily detected in CD206⁺ macrophages in the border of the cortex. Minor immunoreactivity of NLRP3 was also detected in deep parenchyma (not quantified). Scale bar, 80 μm. **b**, Quantification of CD206⁺NLRP3⁺ cell density in the cortical border of WT, *Mecp2*$^{308/Y}$, *P2x7r*$^{-/-}$ and *Mecp2*$^{308/Y}$;*P2x7r*$^{-/-}$ mice (*n* = 4, 5,

4, 5; P = 0.0083, 0.8163, 0.5160). **c**, Quantification of NLRP3$^+$ cell density in the cortical border of WT, *Mecp2*$^{308/Y}$, *P2x7r*$^{-/-}$ and *Mecp2*$^{308/Y}$;*P2x7r*$^{-/-}$ mice (n = 4, 5, 4, 5; P = 0.0017, 0.9996, 0.5730). **d**, Validation of P2X7R knockout in CD45$^+$ cells from *P2x7r*$^{-/-}$ mice. Histograms showing distribution of P2X7R fluorescence intensity in total CD45$^+$ cells obtained from WT, *Mecp2*$^{308/Y}$, *P2x7r*$^{-/-}$ and *Mecp2*$^{308/Y}$;*P2x7r*$^{-/-}$ mouse cortex. Data are presented as mean ± SEM. $^{**}P < 0.01$, ns, not significant, one-way ANOVA followed by Sidak's multiple comparison test in **b** and **c**. Source data are provided as a Source Data file.

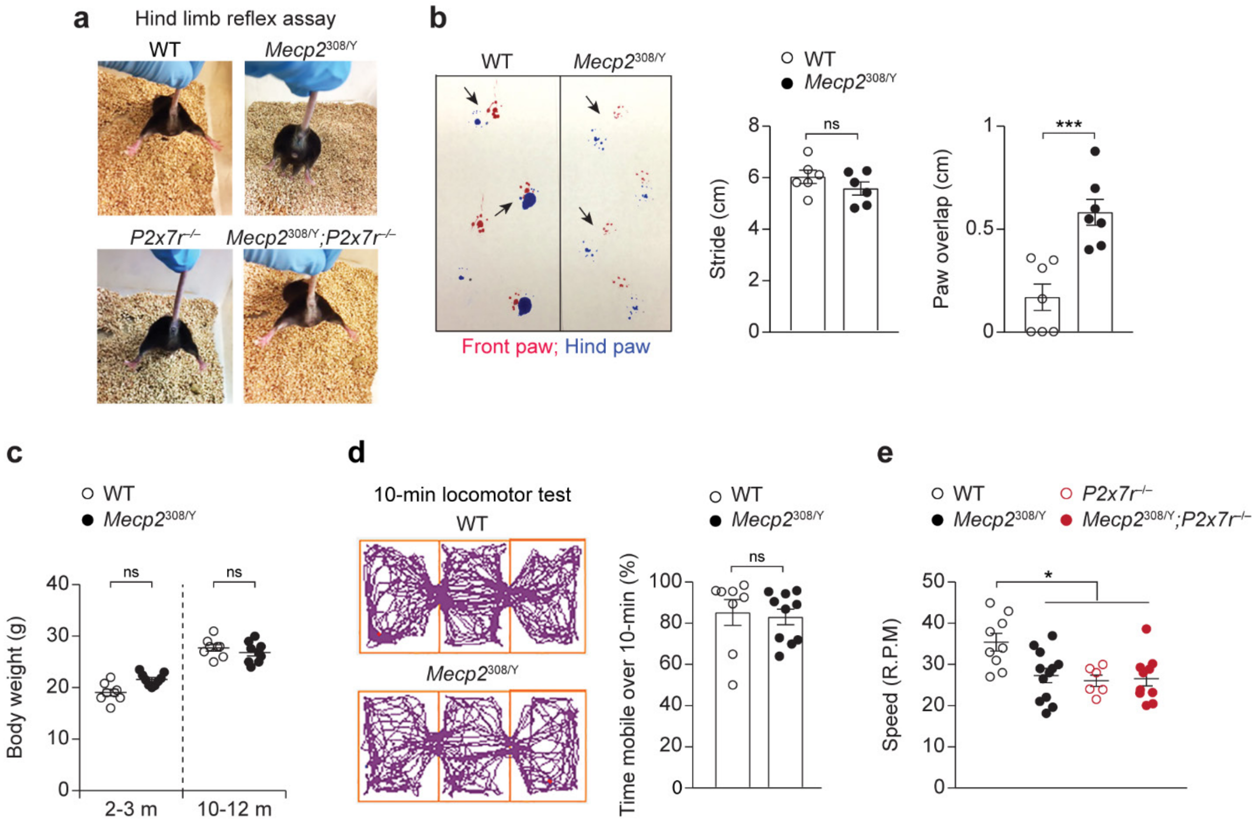

Supplementary Figure 4. Behavioral assessments in *Mecp2*$^{308/Y}$ mice.

a, Representative examples showing absence or presence of clasping reflex in WT, *Mecp2*$^{308/Y}$, *P2x7r*$^{-/-}$ and *Mecp2*$^{308/Y}$;*P2x7r*$^{-/-}$ mice. **b**, Footprint analysis of stride (n = 6 mice per group, P = 0.2363) and paw overlap (n = 7 mice per group, P = 0.0006) in WT and *Mecp2*$^{308/Y}$ mice. **c**, Body weight of WT (n = 8) and *Mecp2*$^{308/Y}$ (n = 9) mice at various ages. **d**, Left: representative trajectories of a WT and *Mecp2*$^{308/Y}$ mouse during a 10-min locomotor test in the 3-chamber arena. Right: Quantification of the animals' mobile time over 10 min (n = 8, 10; P = 0.7586). **e**, Performance of WT (n = 9), *Mecp2*$^{308/Y}$ (n = 12), *P2x7r*$^{-/-}$ (n = 6) and *Mecp2*$^{308/Y}$;*P2x7r*$^{-/-}$ (n = 10) mice in the rotarod test ($F_{3, 33}$ = 5.266, P = 0.0044; *Mecp2*$^{308/Y}$ vs. WT, P = 0.0116; *P2x7r*$^{-/-}$ vs. WT, P = 0.0154; *Mecp2*$^{308/Y}$;*P2x7r*$^{-/-}$ vs. WT, P = 0.0075). Rotarod performance is expressed as the average speed reached during a 40-trial training session. Data are presented as mean ± SEM. *P < 0.05, ***P < 0.001, ns, not significant, unpaired two-tailed t test in **b**-**d**, one-way ANOVA followed by Sidak's multiple comparison test in **e**. Source data are provided as a Source Data file.

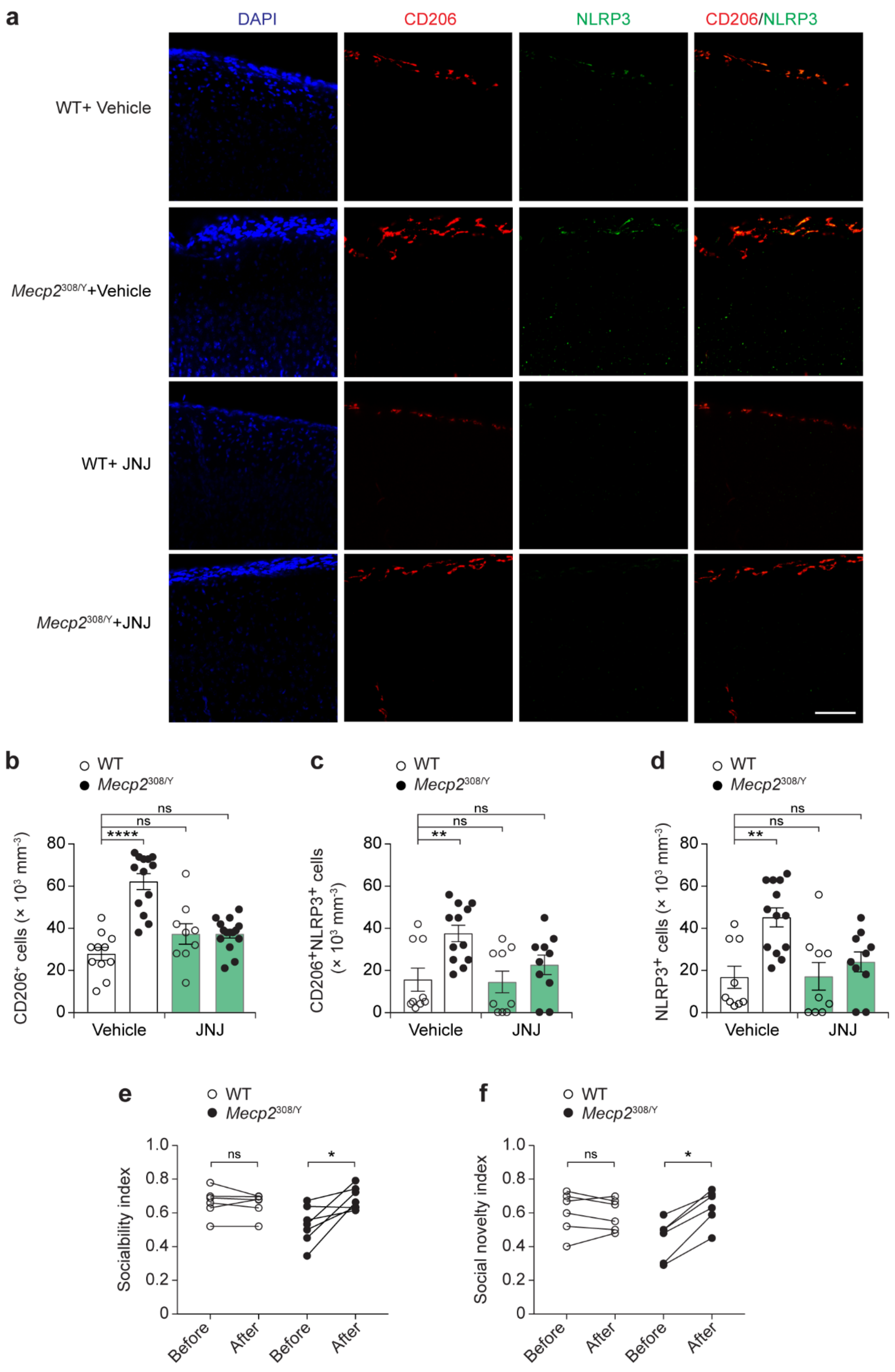
a
DAPI
CD206
NLRP3
CD206/NLRP3
WT+ Vehicle
Mecp2308/Y+Vehicle
WT+ JNJ
Mecp2308/Y+JNJ
b
WT
Mecp2308/Y
CD206+ cells (× 10³ mm⁻³)
Vehicle
JNJ
ns
ns
****
c
CD206+NLRP3+ cells (× 10³ mm⁻³)
**
d
NLRP3+ cells (× 10³ mm⁻³)
**
e
Sociability index
Before
After
*
f
Social novelty index

Supplementary Figure 5. Pharmacological inhibition of P2X7Rs in both circulation and CNS reduces inflammatory cell recruitment and improves social behavior in *Mecp2*$^{308/Y}$ mice.

a, Representative coronal sections of the mouse cortex stained for NLRP3 (green) and CD206 (red). Cell nuclei were colored with DAPI (blue). NLRP3 was mostly detected in CD206^{+} macrophages in the border of the cortex, but not in deep perivascular CD206^{+} macrophages. Minor immunoreactivity of NLRP3 was detected in parenchyma (not quantified). JNJ-47965567 (JNJ) is a BBB-permeable P2X7R antagonist. Scale bar, 80 μm. **b**-**d**, Quantification of CD206^{+} (**b**; *n* = 11, 13, 9, 15; *P* < 0.0001, *P* = 0.2003, 0.1223), CD206^{+}NLRP3^{+} (**c**; *n* = 9, 12, 9, 10; *P* = 0.0059, 0.9981, 0.6754) and NLRP3^{+} (**d**; *n* = 9, 13, 9, 10; *P* = 0.0011, *P* > 0.9999, *P* = 0.7207) cell density in the cortical border of WT and *Mecp2*$^{308/Y}$ mice (5-month-old) treated with vehicle or JNJ-47965567 (10 mg/kg/day, 3–5 days). **e**, Sociability index measured in WT and *Mecp2*$^{308/Y}$ mice (5-month-old) before and after the administration of JNJ-47965567 (10 mg/kg/day for 3–5 days, i.p.). WT, *n* = 6, *P* = 0.4375; *Mecp2*$^{308/Y}$, *n* = 7, *P* = 0.0313. **f**, Social novelty index measured in WT and *Mecp2*$^{308/Y}$ mice before and after JNJ-47965567 treatment (10 mg/kg/day for 3–5 days, i.p.). WT, *n* = 6, *P* = 0.6563; *Mecp2*$^{308/Y}$, *n* = 6, *P* = 0.0313. Data are presented as mean ± SEM. **P* < 0.05, ***P* < 0.01, *****P* < 0.0001, ns, not significant, two-way ANOVA followed by Sidak's multiple comparison test in **b-d,** paired two-tailed Wilcoxon signed rank test was used in **e** and **f**. Source data are provided as a Source Data file.

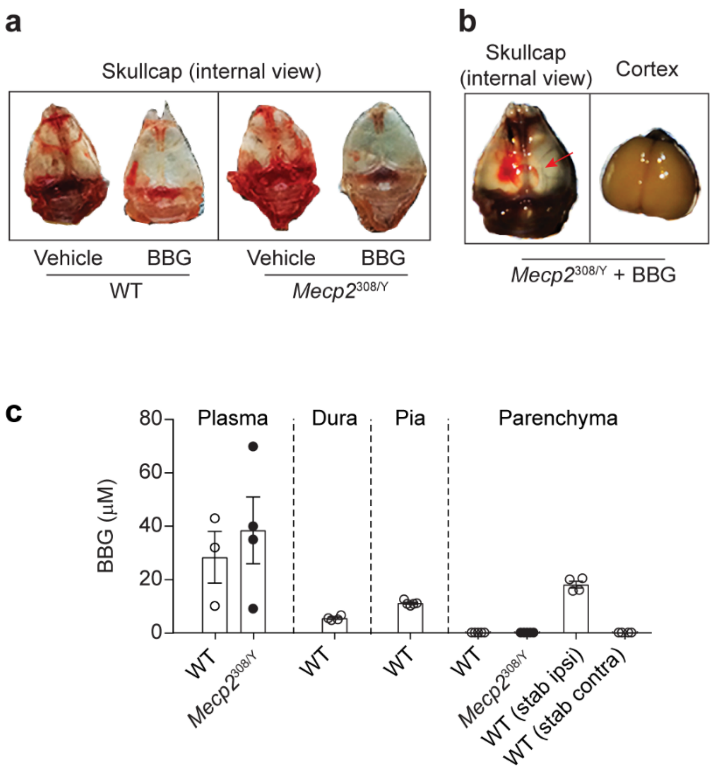

Supplementary Figure 6. Distribution of Brilliant Blue G after intraperitoneal injection.

a, Representative pictures of skullcap from mice treated with vehicle or Brilliant Blue G (BBG) (10 mg/kg/day for 5 days, i.p.). **b**, Left, the internal view of skull and associated dura mater in a *Mecp2*$^{308/Y}$ mouse treated with BBG. Arrow indicates a meningeal vessel turning blue/gray after BBG treatment. Right, the brain from the same mouse after the removal of meninges. **c**, Quantification of BBG concentration in plasma, dura mater, pia mater and cortical parenchyma (*n* = 3, 4, 4, 5, 5 ,5, 4, 4). As a positive control, a stab wound injury (a procedure known to open BBB) was performed in one side of the cortex. Mice received 10 mg/kg BBG for 5 days after injury. BBG was abundant in the injured (ipsilateral) parenchyma, but not detected in the uninjured (contralateral) hemisphere. Data are presented as mean ± SEM. Source data are provided as a Source Data file.

Supplementary Table 1. Cell surface markers used for the identification and characterization of myeloid cells in the brain.

Subset	Cell surface marker
Ly6C^{High} monocytes	CD11b^{+}, CD45^{High}, MHCII^{Low}, Ly6G$^{–}$, CX3CR1^{+}, Ly6C^{High}, CD11c$^{–}$, P2X7R^{Int}
Neutrophils	CD11b^{+}, CD45^{High}, MHCII^{Low}, Ly6G^{+}, Ly6C^{int}, CD11c$^{–}$, P2X7R$^{–}$
CD206^{Low}MHCII^{High} Macrophage	CD11b^{+}, CD45^{+}, Ly6G$^{–}$, Ly6C$^{–}$, CD64^{+}, CD38$^{–}$, MHCII^{High}, CD206^{Low}, P2X7R^{Int}
CD206^{High}MHCII^{Low} Macrophages	CD11b^{+}, CD45^{+}, Ly6G$^{–}$, Ly6C$^{–}$, CD64^{+}, CD38$^{–}$, MHCII^{High}, CD206^{Low}, P2X7R^{High}
Microglia	CD11b^{+}, CD45^{Low}, CD64^{+}, CD38$^{–}$, MHCII^{Low}, CD11c^{Low}, CD206^{Low}, CX3CR1^{High}, P2X7R^{Low}

Supplementary Table 2. Cell surface markers used for the identification and characterization of circulating monocytes and lymphocytes.

Subset	Cell surface marker
Ly6C^{High} monocytes	CD11b^{+}, CD45^{High}, MHCII^{Low}, Ly6G$^{–}$, CX3CR1^{Low}, Ly6C^{High}, CD11c$^{–}$, P2X7R^{High}
Ly6C^{Low} monocytes	CD11b^{+}, CD45^{High}, MHCII^{Low}, Ly6G$^{–}$, CX3CR1^{High}, Ly6C^{Low}, CD11c^{Low}, P2X7R^{High}
Lymphocytes	CD11b$^{–}$, CD45^{High}, P2X7R^{Low}

Supplementary Table 3. Antibodies for flow cytometry and their respective clones.

Antibody to	Supplier	Catalog #	Fluorophore	Clone
CD45	BD Biosciences	564279	BUV395	30-F11
CD11b	BioLegend	101219; 101220	Alexa Fluor 488; Alexa Fluor 647	M1/70
CD11b	ThermoFisher	47-0112-82	APC-eFluor 780	M1/70
CD206	BioLegend	141731;141719; 141711	Pe/Dazzle 594, PE/Cy7, Alexa Fluor 647	C068C2
CD11c	BioLegend	117317	PE/Cy7	N418
Ly6C	BioLegend	128021; 128033	Alexa Fluor 488, Brilliant Violet 510	HK1.4
Ly6G	BioLegend	127615	PerCP/Cy5.5	1A8
MHCII	Biolegend	107643	Brilliant Violet 711	M5/114.15.2
CD64	Biolegend	139309, 139305	Brilliant Violet 421, APC	X54-5/7.1
CD38	Biolegend	102721	PerCP/Cyanine5.5	90
P2X7	Bio-Rad	MCA4713	-	Hano43
