# Supplementary material for: P2X7 receptor inhibition ameliorates dendritic spine pathology and social behavioral deficits in Rett syndrome mice
Source: Nat Commun. 2020 Apr 14;11:1784. doi: 10.1038/s41467-020-15590-5 (PMC7156443; doi:10.1038/s41467-020-15590-5)
Supplement: Supplementary file 3 — Reporting Summary [file 41467_2020_15590_MOESM3_ESM.pdf]

nature research

Corresponding author(s): Guang Yang

Last updated by author(s): 03-12-20

Reporting Summary

Nature Research wishes to improve the reproducibility of the work that we publish. This form provides structure for consistency and transparency in reporting. For further information on Nature Research policies, see Authors & Referees and the Editorial Policy Checklist .

Statistics

For all statistical analyses, confirm that the following items are present in the figure legend, table legend, main text, or Methods section.

n/a	Confirmed	
☐	☒	The exact sample size (*n*) for each experimental group/condition, given as a discrete number and unit of measurement
☐	☒	A statement on whether measurements were taken from distinct samples or whether the same sample was measured repeatedly
☐	☒	The statistical test(s) used AND whether they are one- or two-sided<br>*Only common tests should be described solely by name; describe more complex techniques in the Methods section.*
☐	☒	A description of all covariates tested
☐	☒	A description of any assumptions or corrections, such as tests of normality and adjustment for multiple comparisons
☐	☒	A full description of the statistical parameters including central tendency (e.g. means) or other basic estimates (e.g. regression coefficient) AND variation (e.g. standard deviation) or associated estimates of uncertainty (e.g. confidence intervals)
☐	☒	For null hypothesis testing, the test statistic (e.g. *F*, *t*, *r*) with confidence intervals, effect sizes, degrees of freedom and *P* value noted<br>*Give P values as exact values whenever suitable.*
☒	☐	For Bayesian analysis, information on the choice of priors and Markov chain Monte Carlo settings
☒	☐	For hierarchical and complex designs, identification of the appropriate level for tests and full reporting of outcomes
☒	☐	Estimates of effect sizes (e.g. Cohen's *d*, Pearson's *r*), indicating how they were calculated

Our web collection on statistics for biologists contains articles on many of the points above.

Software and code

Policy information about availability of computer code

Data collection	ANY-Maze 6.0 and EthoVision 11.5: social behavior; EZRod: rotarod; LSRII (BD): flow cytometry; Bruker PrairieView 4.0: Imaging data acquisition
Data analysis	Image J Fiji: imaging analysis; ANY-Maze 6.0 and EthoVision 11.5: social behavior analysis; FlowJo V 10: flow cytometry; GraphPad Prism 7: statistics

For manuscripts utilizing custom algorithms or software that are central to the research but not yet described in published literature, software must be made available to editors/reviewers. We strongly encourage code deposition in a community repository (e.g. GitHub). See the Nature Research guidelines for submitting code & software for further information.

Data

Policy information about availability of data

All manuscripts must include a data availability statement. This statement should provide the following information, where applicable:

- Accession codes, unique identifiers, or web links for publicly available datasets
- A list of figures that have associated raw data
- A description of any restrictions on data availability

Data supporting the findings of this manuscript are available from the corresponding author upon reasonable request. The source data underlying Figs. 1c-d, 1f, 1h-j, 1l, 1n, 2b-h, 3b-i, 4a, 4c, 4e, 5a-f, 6a-h and Supplementary Figs 1b-f, 2c, 3b-c, 4b-e, 5b-f, 6c are provided as a Source Data file.

Field-specific reporting

Please select the one below that is the best fit for your research. If you are not sure, read the appropriate sections before making your selection.

Life sciences study design

All studies must disclose on these points even when the disclosure is negative.

Sample size	No sample-size calculation was performed. The sample sizes were chosen based on previous studies using the same methodologies (Garre et al., 2017 Nat Med 23,714–722; Silva et al., J Exp Med. 2019; 216(4): 786-806) and accepted standards in the field.
Data exclusions	No samples or animals that were successfully imaged or measured were excluded from the analysis.
Replication	No data were excluded from the analysis.
Randomization	All animals were randomly assigned to experimental groups.
Blinding	Investigators were blinded to group allocations during data collection and analysis.

Reporting for specific materials, systems and methods

We require information from authors about some types of materials, experimental systems and methods used in many studies. Here, indicate whether each material, system or method listed is relevant to your study. If you are not sure if a list item applies to your research, read the appropriate section before selecting a response.

Materials & experimental systems

n/a	Involved in the study	
☐	☒	Antibodies
☒	☐	Eukaryotic cell lines
☒	☐	Palaeontology
☐	☒	Animals and other organisms
☒	☐	Human research participants
☒	☐	Clinical data

Methods

n/a	Involved in the study	
☒	☐	ChIP-seq
☐	☒	Flow cytometry
☒	☐	MRI-based neuroimaging

Antibodies

Antibodies used	CD11b (BioLegend #101219; BioLegend #101220; ThermoFisher #47-0112-82; clone M1/70, 0.5ug/ml); CD11c (BioLegend #117317; clone N418, 0.5ug/ml); Ly6C (Biolegend #128021; BioLegend #128033; clone HK 1.4, 0.2ug/ml); CD206 (BioLegend#141731; BioLegend #141719; BioLegend #141711; clone C068C2, 1ug/ml); MHCII (Biolegend #107643; clone M5/114.15.2, 0.1ug/ml); CD64 (BioLegend #139309, #139305; clone x54-5/7.1, 1ug/ml); CD38 (Biolegend #102721; clone 90, 0.5ug/ml); CD45 (BD Biosciences #564279; clone 30-F11, 1ug/ml); Ly6G (BioLegend #127615; clone 1A8, 0.5ug/ml); Iba1 (Wako #019-19741, 1/200); NLRP3 (Abcam #ab4207, 1/200; Abcam #ab214185, 1/100)<br>Please also see Supplementary Table 3.
Validation	Antibodies used in this study have been validated by manufacturer (Biolegend; BD Biosciences; Wako; Abcam), as well as in previous studies (Garre et al., 2017 Nat Med 23,714–722; Silva et al., J Exp Med. 2019; 216(4): 786-806).

Animals and other organisms

Policy information about studies involving animals; ARRIVE guidelines recommended for reporting animal research

Laboratory animals	All mice used in this study were obtained from Jackson Laboratory: C57BL6J (stock no: 000664); Thy1-YFP-H mice (stock no: 003782); Mecp2 308 mice (stock no: 005439); P2x7r–/– mice (stock no: 005576). One to 12-month old male mice were used for all the experiments. Mice were group-housed in temperature-controlled rooms on a 12-h light-dark cycle.
Wild animals	This study did not involve wild animals.
Field-collected samples	This study did not involve samples collected from the field.
Ethics oversight	All animal procedures described in this paper were approved by the Institutional Animal Care and Use Committee (IACUC) of New York University and Columbia University as consistent with National Institutes of Health (NIH) Guidelines for the Care and Use of Laboratory Animals.

Note that full information on the approval of the study protocol must also be provided in the manuscript.

Flow Cytometry

Plots

Confirm that:

- [x] The axis labels state the marker and fluorochrome used (e.g. CD4-FITC).
- [x] The axis scales are clearly visible. Include numbers along axes only for bottom left plot of group (a 'group' is an analysis of identical markers).
- [x] All plots are contour plots with outliers or pseudocolor plots.
- [x] A numerical value for number of cells or percentage (with statistics) is provided.

Methodology

Sample preparation	Methods (section "Preparation of Single-Cell Suspensions from Mouse Tissues and Flow Cytometry")
Instrument	LSRII (BD), Methods (section "Flow Cytometry analysis")
Software	FlowJo V 10, Methods (section "Flow Cytometry analysis")
Cell population abundance	This study did not involve cell sorting.
Gating strategy	Provided in Supplementary Figure 1a and indicated in respective figure legends.

- [x] Tick this box to confirm that a figure exemplifying the gating strategy is provided in the Supplementary Information.
